# Early changes in corticospinal excitability when seeing fearful body expressions

**DOI:** 10.1038/srep14122

**Published:** 2015-09-21

**Authors:** Sara Borgomaneri, Francesca Vitale, Alessio Avenanti

**Affiliations:** 1 Dipartimento di Psicologia, Università di Bologna and Centro studi e ricerche in Neuroscienze Cognitive, Campus di Cesena, Università di Bologna; 47521 Cesena, Italy; 2 IRCCS Fondazione Santa Lucia, 00179 Rome, Italy

## Abstract

Quick inhibition of approach tendencies in response to signals of potential threats is thought to promote survival. However, little is known about the effect of viewing fearful expressions on the early dynamics of the human motor system. We used the high temporal resolution of single-pulse and paired-pulse transcranial magnetic stimulation (TMS) over the motor cortex to assess corticospinal excitability (CSE) and intracortical facilitation (ICF) during observation of happy, fearful and neutral body postures. To test motor circuits involved in approach tendencies, CSE and ICF were recorded from the first dorsal interosseous (FDI), a muscle involved in grasping, and the abductor pollicis brevis (APB), which served as a control. To test early motor dynamics, CSE and ICF were measured 70–90 ms after stimulus onset. We found a selective reduction in CSE in the FDI when participants observed fearful body expressions. No changes in ICF or in the excitability of APB were detected. Our study establishes an extremely rapid motor system reaction to observed fearful body expressions. This motor modulation involves corticospinal downstream projections but not cortical excitatory mechanisms, and appears to reflect an inhibition of hand grasping. Our results suggest a fast visuo-motor route that may rapidly inhibit inappropriate approaching actions.

Fearful expressions signal the presence of potential threats in the environment. Because the source of the danger is not clearly signaled, detecting such expressions in others is thought to enhance sensory vigilance in order to garner more information about threats in the surroundings^1^,^2^. In support of this notion, behavioral studies have shown enhanced sensory acquisition^3^, perceptual processing^4^ and attention^2^,^5^ during exposure to fearful expressions. Moreover, fearful expressions trigger larger early occipito-temporal event-related potential (ERP) components than happy or neutral expressions^6^,^7^,^8^. Besides increasing sensory vigilance for potential threats, viewing fearful expressions may affect the motor system. Indeed, fear is a biologically primitive emotion that is clearly associated with immediate action tendencies beneficial to survival^9^,^10^,^11^,^12^. Despite this, the neurophysiological mechanisms that underlie motor reactions to fearful expressions remain unclear. Human imaging studies have shown that watching others’ fearful facial and body expressions increases activity in subcortical (e.g., amygdala and superior colliculus) and cortical regions (e.g., cingulate cortex and supplementary motor area, SMA) that are known to be involved in emotional processing and motor control^13^,^14^,^15^,^16^ and project directly or indirectly to the spinal cord^17^,^18^. However, the functional meaning of such motor activations remains ambiguous because imaging methods have poor temporal resolution and cannot distinguish between excitatory and inhibitory activity. The action tendencies supposedly evoked by fearful expressions might occur on the order of milliseconds and consist of either the selection or inhibition of specific motor programs. Thus, a technique with high temporal resolution and the ability to distinguish between excitation and inhibition is fundamental to investigating the physiological bases of these fear-related motor modulations.

Transcranial magnetic stimulation (TMS) is a valuable technique for probing the physiology of the motor system with high temporal resolution. Single-pulse or paired-pulse TMS over the motor cortex (M1) induces motor-evoked potentials (MEPs). MEPs provide an instantaneous read-out of the functional state of the motor system at the time the TMS pulse was applied. The amplitude of MEPs induced by single-pulse TMS reflects the net effect of excitatory and inhibitory inputs to the descending corticospinal pathway, providing a measure of corticospinal excitability (CSE) to which both cortical and spinal excitability could contribute^19^. To directly assess modulations of intracortical excitability within M1, pairs of TMS stimuli can be administered through a single coil placed over M1^20^,^21^. In this paired-pulse TMS protocol, a conditioning TMS pulse below the threshold intensity needed to elicit a MEP is followed at short interstimulus intervals (ISIs) by a suprathreshold test TMS pulse eliciting a MEP. At ISIs of 1–5 ms, the conditioning pulse results in a reduction of the MEP elicited by the test pulse (i.e., “short intracortical inhibition”, SICI), while longer ISIs of 7–20 ms produce MEP facilitation (“intracortical facilitation”, ICF). These inhibitory (SICI) and facilitatory (ICF) modulations of MEP amplitude take place at the cortical level without affecting spinal circuits. The SICI and ICF indices are thought to reflect the activation of separate populations of inhibitory GABAergic and excitatory glutamatergic cortical interneurons in M1, respectively, and thus provide reliable measures of motor cortical activations^20^,^21^.

Using such TMS protocols, we have recently tested whether observing fearful body expressions rapidly affects the motor system^22^,^23^,^24^. In one experiment^22^, participants observed and categorized pictures of happy, fearful or emotionally neutral human body postures. During this task, single-pulse TMS was administered over M1 to assess modulations of CSE, and paired-pulse TMS was used to explore mechanisms of ICF and SICI. To assess relatively rapid neurophysiological reactions, we administered test TMS pulses at 100 or 125 ms after stimulus onset and recorded MEPs from the first dorsal interosseous (FDI). We found that seeing fearful postures decreased ICF relative to seeing happy or neutral postures, and this effect was similar across the two time points. In contrast, no change in CSE or SICI was found, suggesting that the suppression of motor responses in response to fearful expressions found at 100–125 ms was specific to excitatory glutamatergic cortical mechanisms and did not influence inhibitory GABAergic cortical mechanisms or descending corticospinal motor pathways. These findings pointed to the cortical counterpart of an early and fear-specific reduction in motor readiness. However, motor excitability was monitored only in the FDI muscle, which is involved in fine motor control of hand grip during grasping^25^,^26^,^27^. Hence, it remains unclear whether early reductions in motor excitability reflect the neurophysiological correlate of a massive immobilization similar to freezing^28^,^29^ or, rather, the tendency to rapidly suppress approaching movements (i.e., grasping) that may be inappropriate in the context of a potential threat^10^,^28^,^30^. Here we sought to further investigate motor dynamics during perception of fearful body language by testing modulation of cortical (ICF) and corticospinal (CSE) excitability at an earlier time window, i.e., at 70–90 ms after presentation of emotional body postures. Testing motor excitability in this temporal window is particularly interesting because this window: i) corresponds to the peak latency of the earliest cortical response to visual stimuli in the primary visual cortex, i.e., the C1 ERP component^31^, and C1 amplitude was modulated by fearful facial expressions in previous research^6^,^32^; ii) represents a phase in which visual stimuli are not fully processed at a conscious level, according to current models of object recognition and visual consciousness^33^,^34^. Consequently, this design may provide insights into the rapid and unconscious processing of emotions.

Because of the evolutionary importance of rapidly reacting to signs of fear, we hypothesized that viewing fearful postures would reduce motor excitability relative to viewing happy and neutral postures. Our paradigm allowed us to test whether such a neurophysiological suppression would occur at a cortical (i.e., ICF) or a corticospinal (i.e., CSE) level. Lastly, to address whether the hypothesized suppressive responses to fearful bodies would consist of a massive and unspecific reduction in motor excitability or selective modulation of muscles involved in approaching movements, we recorded MEPs from the FDI muscle—which has a major role in grasping (i.e., flexing the index finger at the metacarpal joint) and was used in our previous research^22^—and a nearby control muscle, the abductor pollicis brevis (APB), that is less reliably and finely modulated during grasping^25^,^26^,^27^.

## Results

### Changes in CSE during observation of emotional bodies

**Figure 1:**
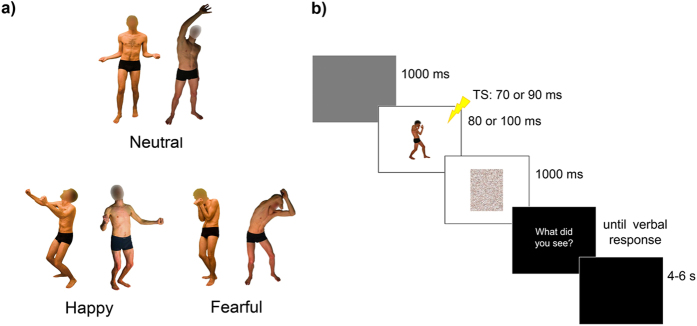
(**a**) Examples of visual body stimuli. (**b**) Trial sequence.

Sixteen participants were tested in an emotion recognition task while receiving TMS over M1 (Fig. 1). They observed pictures of body postures and categorized them as happy, fearful or neutral. TMS pulses eliciting MEPs were administered at 70–90 ms after picture onset. In one session, we assessed changes in CSE induced by the observation of emotional body postures by using single-pulse TMS to elicit MEPs from the FDI and APB muscles. MEP amplitudes in the single-pulse session were analyzed by means of a three-way repeated-measures ANOVA with the factors Muscle (2 levels: FDI and APB), Time (2 levels: 70 and 90 ms) and Posture (3 levels: happy, fearful and neutral).

**Figure 2:**
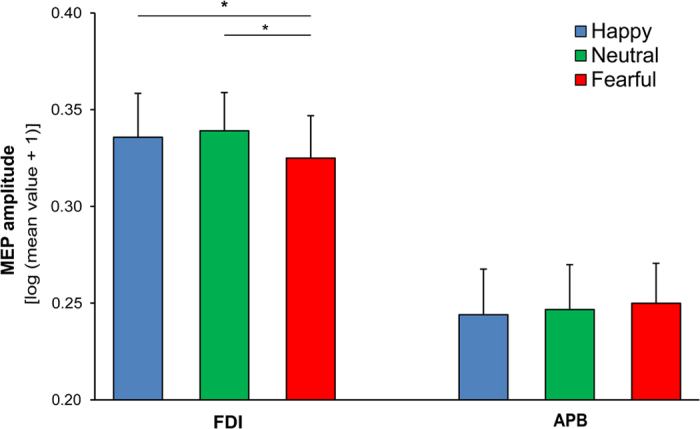
Changes in CSE during the emotion recognition task (single-pulse session). MEP amplitude, recorded from two different hand muscles (FDI and APB) during perception of happy, neutral and fearful body postures (average of the two time points, 70 and 90 ms from stimulus onset). Error bars indicate s.e.m. Asterisks (*) denote significant post-hoc comparisons (p < 0.05).

The Muscle × Time × Posture ANOVA on MEP amplitudes showed a main effect of Muscle (F_1,15_ = 10.26; p = 0.006; eta^2^ = 0.41; greater amplitudes recorded from the FDI relative to the APB muscle) and a main effect of Time (F_1,15_ = 6.66; p = 0.02; eta^2^ = 0.31; greater amplitudes for the 90 ms relative to the 70 ms condition). Critically, a significant Muscle × Posture interaction was found (F_2,30_ = 4.18; p = 0.02; eta^2^ = 0.22; Fig. 2). Post-hoc analyses (Newman-Keuls test) showed that the MEP amplitude recorded from the FDI was lower for fearful postures than for happy (p = 0.03; d = 0.27) and neutral postures (p = 0.02; d = 0.51) which in turn did not significantly differ from one another (p = 0.50). No significant modulations were found in the APB muscle (all p > 0.44). The triple interaction was not significant (F_2,30_ = 1.21; p = 0.31; eta^2^ = 0.07), suggesting that the reduction in FDI excitability for fearful postures was similar in the 70 ms and 90 ms conditions (see Table 1). No other significant effects were found in the ANOVA (all F < 2.92, p > 0.11).

### Changes in ICF during observation of emotional bodies

**Figure 3:**
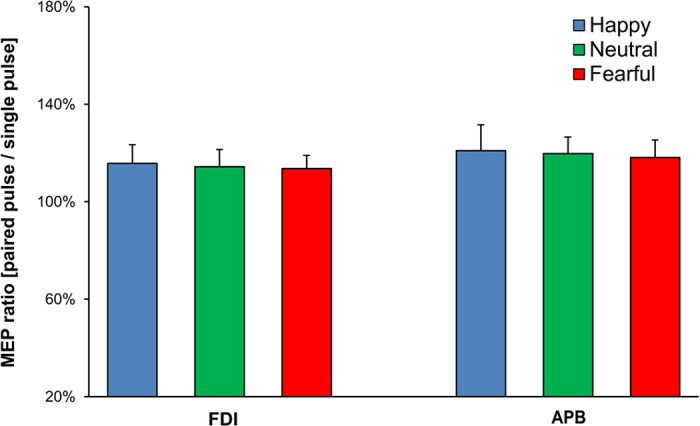
Changes in ICF during the emotion recognition task. MEP amplitude ratio (paired-pulse MEPs divided by single-pulse MEPs), recorded from two different hand muscles (FDI and APB) during perception of happy, neutral and fearful body postures (average of the two time points, 70 and 90 ms from stimulus onset). Error bars indicate s.e.m.

To evaluate changes in ICF, participants performed the same emotion recognition task in an additional session while undergoing an established paired-pulse TMS protocol^20^,^21^ in which a conditioning pulse and a test pulse were delivered through the same coil over M1 (interpulse interval: 12 ms). ICF was assessed by means of MEP ratios computed separately for each condition (mean conditioned MEP in the paired-pulse session relative to mean unconditioned test MEP in the single-pulse session^20^,^21^. This analysis showed no significant main effects or interactions (all F < 2.93, p > 0.07; Fig. 3).

### Behavioral performance in the emotion recognition task

Mean accuracy in the emotion recognition task was high in both TMS sessions (single-pulse TMS mean accuracy ±S.D.: 88.5% ± 5.0; paired-pulse TMS: 88.8% ± 5.5). The Session × Time × Posture ANOVA carried out on accuracy showed no main effects or interactions (all F < 2.74, p > 0.08) suggesting that visual recognition was comparable in the two sessions and across the different conditions.

### Subjective measures

After TMS, participants were presented with all the body stimuli and asked to judge arousal, valence and perceived movement. Ratings were analyzed by means of non-parametric Friedman ANOVAs. The analyses of valence and arousal ratings were significant (all χ^2^ > 18, p < 0.0001). Follow-up comparisons confirmed that valence was more negative for fearful relative to happy and neutral body stimuli (all p < 0.0004); moreover, valence was more positive for happy relative to neutral postures (all p < 0.0004). Arousal for happy and fearful postures was comparable (p = 0.42) and greater than for neutral postures (all p < 0.001). The Friedman ANOVA on implied motion was not significant (χ^2^ = 4.88, p = 0.09), suggesting that the three postures contained similar amounts of implied motion (Table 2).

## Discussion

In this study, we recorded MEPs to investigate changes in CSE and ICF during observation of emotional body postures. MEPs were recorded in an early time window (70–90 ms) to tap fast motor reactions to emotional bodies. We found that seeing fearful body expressions reduced CSE relative to seeing happy or emotionally neutral body postures. This reduction was specific to a muscle involved in grasping (i.e., the FDI) and did not extend to a nearby control muscle (i.e., the APB). These findings suggest that early reactions to fearful body postures involve the inhibition of approach action tendencies.

Our findings provide neurophysiological evidence supporting theoretical models of emotion and emotion perception that postulate a fundamental link between emotions and action tendencies^9^,^10^,^11^,^12^. Emotional body expressions serve an important communicatory role by allowing rapid transmission of emotion information from one individual to another^11^,^16^,^35^. In particular, fearful expressions are thought to communicate potential threats in the surrounding environment^1^,^2^,^3^,^4^,^5^, and thus it is assumed that detecting such expressions would prepare the organism to rapidly inhibit approach tendencies. However, few studies have investigated early neurophysiological modulations in the human motor system when processing fearful expressions.

In a recent series of TMS studies, we started to investigate neurophysiological changes in the motor system during observation of emotional body postures^22^,^23^,^24^. We explored motor responses in the 100–300 ms time window and found that viewing emotional bodies indeed led to inhibitory modulations when motor excitability was tapped at 100–150 ms after stimulus onset^22^,^24^. Stronger motor suppression for fearful body postures was selectively found in the 100–125 ms time window. However, the nature of the inhibitory modulation was unclear, as MEPs were recorded from a single muscle, i.e., the FDI. In principle, early motor inhibition in response to fearful body expressions could reflect two possible mechanisms: i) a massive reduction in motor output consisting of a freezing-like immobility that may affect the upper limb or even the whole body, and could promote monitoring for the source of danger^28^,^29^; or ii) a more organized response, consisting of the selective inhibition of action tendencies that are inappropriate when facing potential threats, i.e., approaching movements like grasping^10^,^28^,^30^. Notably, the latter mechanism, but not the former, predicts that motor inhibition should be specific for those muscles involved in grasping, e.g., the FDI. Our study suggests that in an early time window, i.e., 70–90 ms after stimulus onset, the motor system implements muscle-specific motor responses that might reflect inhibition of approach tendencies, as they mostly involved the FDI—a muscle implicated in grasping—and not a control muscle.

The early timing of our CSE modulations appears in keeping with previous studies. For example, Cantello and colleagues^36^ showed that unexpected visual flashes reduced CSE at 55–70 ms after stimulus onset. Moreover, Makin and colleagues^37^ reported that the sudden appearance of an object rapidly approaching the body reduced CSE with a latency of 70–80 ms. Our study significantly expands these previous findings by showing that, in a similar early temporal window, the brain can discriminate much more complex visual stimuli such as human body postures, extract emotion information from them and inhibit non-adaptive motor responses (i.e., hand approach) with fine-grained muscle specificity.

In this study, we selected the FDI and APB muscles because they were commonly used in previous TMS studies on emotion perception^38^,^39^,^40^ and showed similar CSE modulations for emotional scenes and bodies when MEPs were measured at 150–300 ms after stimulus onset^23^,^41^. On the other hand, the FDI, but not the APB, has a major role in controlling grip during grasping (in particular, in controlling flexion of the index finger^25^,^26^), which is the most common and functional approach movement in the human motor repertoire. The APB is involved in thumb abduction. It has no clear role in grasping because its pulling direction does not allow direct grip force production, suggesting it may instead have a general stabilizing role^25^,^26^,^27^. Therefore, the FDI and APB muscles are ideal for discriminating between the two proposed mechanisms underlying observational fear-related early motor inhibition, i.e., massive freezing-like immobilization vs. inhibition of approach/grasping. Our findings support the latter inhibitory mechanism and allow us to clarify its neurophysiological implementation in the human motor system.

In particular, we found that suppression of the FDI muscle when viewing fearful postures occurred at the corticospinal level (CSE) but not the intracortical level (ICF). This indicates that fearful body expressions quickly affect downstream projections to the spinal cord but do not influence M1, or at least the glutamatergic excitatory M1 circuits tapped by the ICF index. Since we did not measure SICI or other neurophysiological indices of inhibition, we cannot exclude that cortical inhibitory networks may be involved in the suppression of CSE. However, the level at which motor suppression occurred in the present experiment (i.e., the corticospinal system) clearly differs from the level implicated in a previous TMS study^22^. In that study, we tested CSE, ICF and SICI by recording MEPs from the FDI at 100–125 ms after stimulus onset. The results showed a reduction in ICF for fearful relative to happy and neutral body postures, but no modulations of CSE or SICI^22^. Taken together, our previous findings and the present study suggest two different steps in the early motor response to fearful bodies: an initial suppression of corticospinal (but not cortical) excitability (70–90 ms) which may reflect modulations of spinal excitability, followed by cortical (but not corticospinal) suppression (100–125 ms). The earlier CSE modulation that we have reported here may have greater functional relevance for action execution than the later selective modulation of glutamatergic mechanisms that we previously reported^22^, because only the former consists of a reduction in corticospinal motor output.

At present, we can only speculate about whether these two modulations reflect two phases of the same mechanism (i.e., the inhibition of approach tendencies) or two functionally distinct processes (e.g., an early inhibition of action tendencies at about 70–90 ms, followed by a generalized reduction in the propensity to move the body at about 100–125 ms). Further studies are needed to test the muscle specificity of the fear-related suppressive effects in both temporal windows and the relationship between the two effects.

The issue of whether the two suppressive effects reflect similar or distinct mechanisms is also related to the question of whether these motor modulations are supported by similar or distinct neural pathways. In principle, visual information about emotional bodies can be conveyed via a subcortical or cortical route^42^,^43^,^44^,^45^. Studies have suggested that visual processing of affective stimuli could influence motor output via subcortical pathways bypassing the cortex^9^,^46^. Imaging evidence indicates that perception of emotional bodies activates subcortical structures (i.e., pulvinar, caudate nucleus and amygdala^16^,^47^) even in cortically blind patients with damage to the striate cortex^48^, suggesting that subcortical structures receive inputs from the retina that bypass the damaged visual cortex. Notably, these structures also possess downstream projections influencing the spinal cord^17^,^18^. Therefore, the fast modulation of CSE (but not ICF) that we found in the present study may reflect a rapid inhibition of approaching movements through a mainly subcortical route, influencing downstream projections to the spinal cord. On the other hand, later cortical motor responses to fearful bodies (i.e., the ICF modulation we found in our previous study^22^) may occur through the activation of a cortical route from visual to parietal and frontal areas involved in action execution^16^,^49^,^50^,^51^. This interpretation would fit the well-established notion that emotional processing involves an initial rapid activation of subcortical routes for fast motor reactions and slower cortical routes for more refined responses^9^,^52^. Although this hypothesis is plausible and grounded in the literature, other possibilities cannot be ruled out. For example, subcortical regions that are active when seeing emotional bodies also possess upstream projections influencing the cortical motor system^17^,^18^. Therefore, the ICF modulation we previously reported^22^ could be implemented through a mainly subcortical or cortico-subcortical pathway, ultimately projecting to M1. Additionally, we do not rule out that more direct occipito-frontal connections^53^,^54^ (e.g., inferior fronto-occipital fascicle) could also contribute to the rapid CSE modulation we report here.

One potential methodological issue is that our stimulation protocol may have been optimized for recording MEPs from the FDI muscle. We find it unlikely, however, that the lack of APB modulation is due to such a technical factor. Indeed, in our previous TMS studies we recorded MEPs from the FDI and the APB during observation of emotional bodies and emotional scenes, and used the same stimulation protocol as in the present experiment^23^,^41^. Despite these similarities, in the previous studies we found a comparable change in CSE in the two muscles when MEPs were recorded at 150–300 ms from stimulus onset (and thus reflected a different process than the one found in the present study). Thus, the topographic specificity of our findings appears to reflect a specific early mechanism (i.e., the inhibition of action tendencies) rather than an artifact of the recording procedure. However, an important goal for future research would be to acquire MEPs from additional muscles. For example, testing additional muscles involved in grasping (i.e., finger flexors) and other control muscles may provide convergent support to our hypothesis. In a similar vein, because grasping is considered an approaching movement related to reaching, future studies could test whether motor representations of muscles typically involved in reaching (i.e., deltoids) are also inhibited when observing fearful bodies. While our data suggest an inhibition of approaching hand movements, one should also consider that behavioral responses to fear typically include fight/flight reactions^12^,^28^,^29^,^52^,^55^. Our data do not rule out that these reactions could also be rapidly implemented in the motor system when seeing fearful bodies. Indeed, it might be that these reactions unfold at different time points, or are implemented simultaneously but by different motor circuits and muscles not sampled in our study (e.g., leg muscles). Similarly, while neural activity in the motor representation of the hand suggests an inhibition of grasping, testing more proximal segments of the motor system (e.g., the trunk) could better reveal the implementation of freezing-like reactions in response to fearful bodies. Future TMS studies could directly test these different possibilities.

In conclusion, our study shows that watching fearful body language reduces the excitability of corticospinal projections to muscles that are involved in hand grasping in an early time window (70–90 ms), thus suggesting that fear detection involves an early inhibition of approach tendencies.

## Methods

### Participants

Sixteen healthy subjects took part in the study (8 men, mean age ± S.D.: 24.0 y ± 1.4). All participants were naïve to the purpose of the experiment and gave written informed consent before participation. The experimental protocol was approved by the ethics committee of the University of Bologna and was carried out in agreement with legal requirements and international norms (Declaration of Helsinki, 1964). The methods carried out in this work are in accordance with the approved guidelines. Participants were right-handed and free of any contraindications to TMS. No discomfort or adverse effects during TMS were reported or noticed.

### Visual stimuli

Pictures were presented on a 19-inch screen located about 80 cm away from the participant. Forty-five pictures depicting four actors in emotional and neutral postures (Fig. 1a) were selected from a validated database^22^,^23^,^24^. To focus specifically on body-related information, the face was blanked out in all pictures. Stimuli included 15 pictures of happy postures, 15 pictures of fearful postures and 15 pictures of neutral postures with perceived (implied) motion comparable to that of the emotional body expressions but without emotional meaning. The stimuli were well recognized as prototypical representations of the different postures and consisted of whole body movements with a clear involvement of both left and right hands (see^23^ for details).

To rule out the possibility that changes in right M1 excitability might be due to differing amounts of implied motion in the models’ left or right body parts, mirror-reflected copies of the stimuli were also created. Half the participants were tested with the original version of the stimuli, while the remaining participants were tested with mirror-reflected copies. Preliminary analyses showed no effect of stimulus set on MEPs or subjective ratings, so data from the two subgroups of participants were merged.

### TMS and electromyography (EMG) recording

MEPs induced by TMS of the right M1 were recorded from the left FDI and APB muscles with a Biopac MP-35 (Biopac, U.S.A.) electromyograph. EMG signals were band-pass filtered (30–500 Hz), sampled at 5 kHz, digitized and stored on a computer for off-line analysis. Pairs of silver-chloride surface electrodes were placed in a belly-tendon montage over the two muscles with ground electrodes on the left wrist. A figure-of-eight focal coil was connected to a Magstim Bistim^2^ stimulator (Magstim, U.K.). The coil was placed tangentially to the scalp at a 45° angle to the mid-line to induce a posterior–anterior current flow across the central sulcus. The hand motor area of the right M1 was defined as the point where stimulation consistently evoked the largest MEP in the FDI. From that position a stable signal could also be recorded from the APB. We defined the resting motor threshold (rMT) as the lowest intensity that evoked 5 small responses (~50 μV) in the relaxed FDI muscle in a series of 10 stimuli^56^. MEPs were recorded in two counterbalanced single-pulse and paired-pulse sessions. During the single-pulse session, intensity was set to evoke MEPs with a peak-to-peak amplitude of ~1.0 mV. During the paired-pulse session, we assessed ICF using an established protocol^20^,^21^ in which the conditioning pulse and test pulse were delivered through the same coil with an interstimulus interval of 12 ms. The intensity of the conditioning pulse was 80% of the rMT. The intensity of the test pulse was the same as that used in the single-pulse session.

### Procedure

The experiment was programmed using Matlab software to control picture presentation and trigger TMS. MEPs were collected in two 90-trial sessions (single-pulse and paired-pulse) in which participants performed an emotion recognition task: they were presented with a picture of a body posture and asked to categorize it as happy, fearful or neutral. As in our previous research^22^,^23^,^24^,^41^ we used an active task to maximize the chance of detecting emotion-specific modulations^38^,^57^. Trial sequence was as follows: a gray screen (1 s duration) indicated the beginning of the trial, followed by the test picture presented at the center of the screen (Fig. 1b). In half the trials, pictures were presented for 80 ms and a single-pulse (or a test pulse in the paired-pulse session) was administered at 70 ms after stimulus onset. In the remaining trails, pictures were presented for 100 ms and the single-pulse/test pulse was administered at 90 ms after stimulus onset. With these different picture durations we could relate indices of motor excitability with stimulus visibility and discard MEPs associated with incorrect recognition (see data analysis).

Stimulus duration was randomly distributed in the two blocks, and block order was counterbalanced across participants. The picture was followed by a random-dot mask (obtained by scrambling the sample stimulus with image segmentation software) lasting 1 s. Then the question “What did you see?” appeared on the screen, and participants provided a verbal response (forced choice: happy, fear or neutral). To avoid changes in CSE due to the verbal response^58^, participants were instructed to answer about 2–3 seconds after the TMS pulse^59^,^60^. Then, the screen appeared black for 4–6 s, ensuring an inter-pulse interval greater than 10 s. To reduce the initial transient-state increase in motor excitability, before each session two single-pulse (or two paired-pulse stimuli) were delivered over M1 (inter-pulse interval >10 s).

After TMS, participants viewed all the stimuli again (in a randomized order) and judged arousal, valence and perceived movement using a 5-point Likert scale. Each rating was collected separately during successive presentations of the whole set of stimuli.

### Data analysis

Mean MEP amplitude in each condition was measured peak-to-peak (in mV). MEPs associated with incorrect answers (~11%) were discarded from the analysis. Thus, CSE and ICF reflected indices of motor excitability associated with accurate perception of body postures. MEPs with preceding background EMG deviating from the mean by more than 2 S.D. were removed from further analysis (~6%). A logarithmic transformation was applied to the amplitude values [log (mean MEP amplitude value +1)] to normalize the data distribution. To assess changes in CSE, log transformed MEP amplitudes in the single-pulse session were analyzed. To assess ICF, we expressed MEPs from the paired-pulse session relative to those from the single-pulse session (to estimate the effects of the subthreshold conditioning pulse on the MEP elicited by the suprathreshold test pulse). For each experimental condition, we calculated the ratio of the mean conditioned MEP over the mean unconditioned test MEP^20^,^21^. A Muscle × Time × Posture ANOVA was used to analyze CSE and ICF. Accuracy in the emotion recognition task was analyzed by means of a Session × Time × Posture ANOVA. In all the ANOVAs, post-hoc comparisons were carried out with Newman-Keuls tests. Partial eta^2^ was computed as a measure of effect size for the main effects and interactions, whereas repeated measures Cohen’s d was computed for post-hoc comparisons.

Mean VAS ratings for arousal, valence and perceived (implied) movement were not normally distributed (as shown by the Shapiro-Wilk test) and thus were analyzed by means of nonparametric Friedman ANOVAs and Bonferroni-corrected Wilcoxon matched pairs tests.

## Additional Information

**How to cite this article**: Borgomaneri, S. *et al.* Early changes in corticospinal excitability when seeing fearful body expressions. *Sci. Rep.* **5**, 14122; doi: 10.1038/srep14122 (2015).

## Figures and Tables

**Table 1:** Mean (± standard deviation) MEP amplitude recorded at 70 ms and 90 ms after presentation of happy, neutral and fearful body postures (single-pulse session).

	**70 ms**			**90 ms**		
	**Happy**	**Neutral**	**Fearful**	**Happy**	**Neutral**	**Fearful**
FDI	0.333 ± 0.09	0.321 ± 0.08	0.319 ± 0.08	0.338 ± 0.10	0.357 ± 0.10	0.331 ± 0.09
APB	0.241 ± 0.09	0.240 ± 0.09	0.250 ± 0.10	0.247 ± 0.11	0.253 ± 0.09	0.250 ± 0.08

**Table 2:** Mean (± standard deviation) subjective evaluations (arousal, valence and perceived implied motion) of the body stimuli.

	**Happy**	**Neutral**	**Fearful**
Arousal	3.69 ± 0.65	2.10 ± 0.42	3.45 ± 0.81
Valence	4.46 ± 0.30	3.19 ± 0.16	1.46 ± 0.30
Perceived motion	3.39 ± 0.58	3.04 ± 0.60	3.14 ± 0.87
